# Automatic Classification Between COVID-19 and Non-COVID-19 Pneumonia Using Symptoms, Comorbidities, and Laboratory Findings: The Khorshid COVID Cohort Study

**DOI:** 10.3389/fmed.2021.768467

**Published:** 2021-11-18

**Authors:** Hamid Reza Marateb, Farzad Ziaie Nezhad, Mohammad Reza Mohebian, Ramin Sami, Shaghayegh Haghjooy Javanmard, Fatemeh Dehghan Niri, Mahsa Akafzadeh-Savari, Marjan Mansourian, Miquel Angel Mañanas, Martin Wolkewitz, Harald Binder

**Affiliations:** ^1^The Biomedical Engineering Department, Engineering Faculty, University of Isfahan, Isfahan, Iran; ^2^Department of Electrical and Computer Engineering, University of Saskatchewan, Saskatoon, SK, Canada; ^3^Department of Internal Medicine, School of Medicine, Isfahan University of Medical Sciences, Isfahan, Iran; ^4^Department of Physiology, Applied Physiology Research Center, School of Medicine, Cardiovascular Research Institute, Isfahan University of Medical Sciences, Isfahan, Iran; ^5^School of Medicine, Isfahan University of Medical Sciences, Isfahan, Iran; ^6^Isfahan Clinical Toxicology Research Center, Isfahan University of Medical Sciences, Isfahan, Iran; ^7^Automatic Control Department (ESAII), Biomedical Engineering Research Centre (CREB), Universitat Politècnica de Catalunya-Barcelona Tech (UPC), Barcelona, Spain; ^8^Department of Epidemiology and Biostatistics, School of Health, Isfahan University of Medical Sciences, Isfahan, Iran; ^9^Biomedical Research Networking Center in Bioengineering, Biomaterials, and Nanomedicine (CIBER-BBN), Madrid, Spain; ^10^Faculty of Medicine and Medical Center, Institute of Medical Biometry and Statistics, University of Freiburg, Freiburg, Germany

**Keywords:** COVID-19, computer-aided diagnosis, screening, validation studies, machine learning

## Abstract

Coronavirus disease-2019, also known as severe acute respiratory syndrome coronavirus 2 (SARS-CoV-2), was a disaster in 2020. Accurate and early diagnosis of coronavirus disease-2019 (COVID-19) is still essential for health policymaking. Reverse transcriptase-polymerase chain reaction (RT-PCR) has been performed as the operational gold standard for COVID-19 diagnosis. We aimed to design and implement a reliable COVID-19 diagnosis method to provide the risk of infection using demographics, symptoms and signs, blood markers, and family history of diseases to have excellent agreement with the results obtained by the RT-PCR and CT-scan. Our study primarily used sample data from a 1-year hospital-based prospective COVID-19 open-cohort, the Khorshid COVID Cohort (KCC) study. A sample of 634 patients with COVID-19 and 118 patients with pneumonia with similar characteristics whose RT-PCR and chest CT scan were negative (as the control group) (dataset 1) was used to design the system and for internal validation. Two other online datasets, namely, some symptoms (dataset 2) and blood tests (dataset 3), were also analyzed. A combination of one-hot encoding, stability feature selection, over-sampling, and an ensemble classifier was used. Ten-fold stratified cross-validation was performed. In addition to gender and symptom duration, signs and symptoms, blood biomarkers, and comorbidities were selected. Performance indices of the cross-validated confusion matrix for dataset 1 were as follows: sensitivity of 96% [confidence interval, CI, 95%: 94–98], specificity of 95% [90–99], positive predictive value (PPV) of 99% [98–100], negative predictive value (NPV) of 82% [76–89], diagnostic odds ratio (DOR) of 496 [198–1,245], area under the ROC (AUC) of 0.96 [0.94–0.97], Matthews Correlation Coefficient (MCC) of 0.87 [0.85–0.88], accuracy of 96% [94–98], and Cohen's Kappa of 0.86 [0.81–0.91]. The proposed algorithm showed excellent diagnosis accuracy and class-labeling agreement, and fair discriminant power. The AUC on the datasets 2 and 3 was 0.97 [0.96–0.98] and 0.92 [0.91–0.94], respectively. The most important feature was white blood cell count, shortness of breath, and C-reactive protein for datasets 1, 2, and 3, respectively. The proposed algorithm is, thus, a promising COVID-19 diagnosis method, which could be an amendment to simple blood tests and screening of symptoms. However, the RT-PCR and chest CT-scan, performed as the gold standard, are not 100% accurate.

## Introduction

Among all infectious diseases known to humankind that caused massive pandemics, certainly, COVID-19 has been the most recognized one. Despite all the efforts and experiences in fighting these contagious diseases in the past, we are still struggling to control the situation globally. Almost all epidemics were resolved in the past, but their damages were long-standing (1). According to the World Health Organization (WHO), more than 180 million people have been diagnosed, and nearly 4 million souls have been lost since the outbreak of COVID-19 in December 2019 until June 2021. The spread of the virus has not been consistent, and it is mutating rapidly, making it even harder to confront. Fortunately, vaccines came to our aid to eradicate the virus at once. However, only 2.5 billion doses have been administered. Therefore, this pandemic is still ravaging some parts of the world. Also, lockdowns and restrictions have reviled or created fundamental problems in our daily lives, such as undiscovered market patterns and transitions in short-term economic strategies (2). On the whole, an increase in the number of mental health problems was reported in some countries during the COVID-19 pandemic (3). In the future, to prevent the widespread of such contagious airborne diseases, an agile diagnosis will be crucial.

Coronavirus disease-2019 is a severe type of pneumonia (4); symptoms of various types of pneumonia infections are very similar, and it is almost impossible for physicians to diagnose COVID-19 without proper means of examination. There are two viral methods to determine whether the virus infects someone or not; the first is using chest computerized tomography (CT) images (4), and the second is reverse-transcription polymerase chain reaction (RT-PCR) test (5), which is based on respiratory samples of a patient, like nasal mucus. The CT scan is not mobile, and multiple scans magnify any risk of getting cancer, although currently, RT-PCR kits are much more available publicly. Thus, the gold standard for COVID-19 diagnosis is RT-PCR. However, viral RT-PCR has limited sensitivity and may need up to 48 h because of technical dilemmas (6).

Many blood factors, along with body symptoms, can expose the illness. These factors are significantly differentiable between healthy and hospitalized individuals, like D-dimer, alanine transaminase (ALT), C-reactive protein (CRP), bilirubin, lymphocytes, platelets, albumin, neutrophils, diastolic blood pressure, heart rate levels, and Charlson Comorbidity Index (7–14). Furthermore, lactate dehydrogenase (LDH) and α-hydroxybutyrate dehydrogenase (α-HBDH) levels were two noticed markers that discriminate COVID-19 from other kinds of pneumonia. Also, liver functionality was altered considerably (4).

It is worth mentioning that RT-PCR is not always reliable, and that it has its limitations. Long et al. demonstrated that the sensitivity of the RT-PCR test is only 83.3% (15); in addition, RT-PCR standalone diagnosis showed false positive outcomes (16, 17). Interpretation of blood test results does not require dedicated testing kits, and it can be performed in any laboratory. Therefore, a method that can accurately detect COVID-19 would be more favorable, especially in countries with a shortage of RT-PCR reagents and proper laboratories. In this scenario, machine learning and automatic diagnosis combined with blood test results play a vital role. Accordingly, routine blood exams, with symptoms of patients, could diagnose the SARS-CoV-2 infection with an accuracy of above 82%; these factors can, in fact, separate COVID-19 from other kinds of pneumonia (18).

Most computer-aided diagnosis (CAD) studies with blood examinations only considered COVID-19 cases vs. healthy ones and ignored other pneumonia types (11, 19–21). Further studies illustrated that various kinds of pneumonia diseases had similar characteristics (4); hence, without considering this fact, automatic COVID-19 detection algorithms may have a substantial pragmatic bias, and the number of misclassified cases rises. On the other hand, a few CT and x-ray CAD systems distinguished COVID-19 from other types of pneumonia (5, 20, 22–27).

### Related Studies

Several studies analyzed the possibility of diagnosing COVID-19 from blood tests by combining blood tests and symptoms. Goodman-Meza et al. (19) used the UCLA electronic health record data and developed a CAD system for hospital settings. They included complete blood counts and an inflammatory marker (LDH) with the gold standard PCR test. They used an ensemble learning method with several renowned machine learning algorithms [e.g., support vector machine (SVM), logistic regression, multi-level perceptron (MLP) neural network, stochastic gradient descent, and extreme gradient boosting (XGBOOST)], along with hold-out validation method (1,455 and 392 samples for training and test sets), and achieved an area under ROC (AUC) of 0.83.

Zoabi et al. (21) investigated the nationwide public data reported by the Israeli Ministry of Health, in which eight binary symptomatic features (the appearance of five initial clinical symptoms, known contact with an infected individual, sex, and age ≥60) were presented along with the result of the RT-PCR test (nasopharyngeal swab11) as the ground truth. With the hold-out evaluation method (51,831 and 47,401 samples for training and test sets), they achieved an AUC of 0.86 using a light gradient-boosting machine (LightGBM) learning algorithm based on a decision tree (28). Their dataset contained healthy control and patients with COVID-19.

Banerjee et al. (29) obtained the AUC of 0.94 by stratified 10-fold cross-validation on 598 individuals diagnosed with different kinds of pneumonia, despite others, without symptoms or history of the patients. They utterly relied on blood test results and the gold standard RT-PCR test. Their approach was based on three classifiers: random forest decision tree, MLP neural network, and Lasso-elastic-net regularized generalized linear models.

Feng et al. (30) investigated the data of 132 healthy individuals and patients with COVID-19 containing information like vital signs, epidemiological history of exposure, comorbidities, blood routine values, and clinical symptoms. They used Adaboost, LASSO, logistic regression with ridge regularization decision Tree, and 10-fold cross-validation, and achieved an AUC of 0.84.

Wu et al. (31) examined 169 patients from multiple sources (originally from Lanzhou city), from which 27 subjects had COVID-19. Moreover, these patients were diagnosed with different kinds of respiratory-related diseases (e.g., tuberculosis and lung cancer). Based on the hematological and biochemical parameters of their patients and a random forest decision tree with 10-fold cross-validation, an AUC of 0.99 was obtained.

While CT scan machines and RT-PCR kits are not available everywhere, blood tests are widely available and faster. Most of the previous studies have ignored other types of pneumonia infections even though not all patients with COVID-19 symptoms are infected; therefore, presumably, patients may receive wrong medications based on previous methods. Our goal here is to propose a reliable and practical method with a suitable dataset.

This study described a clinically reliable CAD system for distinguishing COVID-19 pneumonia from other types of pneumonia and healthy individuals using blood tests, symptoms, and comorbidities. The discrimination between COVID-19 and distinct types of pneumonia is not a trivial task, as these airborne diseases show similar symptoms (4). Hence, our research was based on the Khorshid COVID Cohort (KCC) study (32), which covered different kinds of pneumonia. Additionally, we used the dataset from the studies presented by Zoabi et al. (21, 33) and Banerjee et al. (29) for cross-validation and discrimination between COVID-19 and healthy controls. Such datasets were also used to compare our method with the state-of-the-art.

## Materials and Methods

This study presented a rapid COVID-19 detection system, which is a clinically reliable model to be used in non-clinical settings, based on blood test results and symptoms. Our methodology consisted of feature selection, over-sampling, and ensemble machine learning techniques. The model was developed and evaluated on three datasets, described in the following section.

### Datasets

We used three different sets of data, one containing a national dataset including patients with COVID-19 and non-COVID-19 pneumonia [KCC study (32)], and its combinations with two other public datasets (33, 34).

The first dataset was from the Khorshid COVID-19 Cohort (KCC) study (32, 35, 36). KCC is a hospital-based surveillance study to investigate COVID-19 and non-COVID pneumonia patients since February 2020. Patient recruitment ended in late August 2020. During this period, Khorshid Hospital in Isfahan was the hot outbreak zone in central Iran.

Patients were diagnosed according to the WHO provisional advice (37). Then, those with positive PCR or compatible chest computed high-resolution CT (HRCT) were enrolled as the case group, while patients with non-COVID pneumonia were recruited as the control group. The study was conducted in two parts: admission until discharge or death and follow-ups after hospital discharge.

Demographics, medical history, underlying chronic diseases, socioeconomic status (SES), Charlson Comorbidity Index (CCI), signs, symptoms, chest computed tomographic (CT) scans, laboratory findings, and treatments in the hospital were collected for the control and case groups. On the whole, 55% of all 630 closely observed patients with COVID-19 died (36).

The ethics committee of the Isfahan University of Medical Sciences (IUMS) and other national authorities approved the experimental protocol conforming to the Declaration of Helsinki: [longitudinal epidemiologic investigation of the characteristics of patients with coronavirus infection referring to Isfahan Khorshid Hospital: IR.MUI.MED.REC.1399.029 (https://ethics.research.ac.ir/EthicsProposalViewEn.php?id=127640), and modeling of incidence and outcomes of COVID-19: IR.MUI.RESEARCH.REC.1399.479 (https://ethics.research.ac.ir/EthicsProposalViewEn.php?id=158927)]. The entire subjects gave written informed consent to the experimental procedure. It was given by the first relative family of patients with severe conditions. No minors participated in our study. The sample dataset was provided as follows on https://figshare.com/s/fe311d566d580197cdf1.

The following blood markers were recorded: alanine aminotransferase (ALT), alkaline phosphatase (ALP), aspartate aminotransferase (AST), lactic dehydrogenase (LDH), C-reactive protein (CRP), urea, platelet (PLT), neutrophils (Neut), lymphocytes (Lymph), and white blood cell (WBC) counts, Sodium (Na), hemoglobin, blood urea nitrogen (BUN), potassium (K), blood creatinine (Cr), potassium (P), blood calcium (Ca), and magnesium (Mg). In dataset 1, baseline blood tests for patient with COVID-19 (case) and non-COVID-19 pneumonia (control) were used, in addition to demographics, SES, medical history, and underlying chronic diseases.

We created the second dataset by mixing the 634 subjects with COVID-19 to KCC with randomly selected 634 healthy subjects from the Israeli Ministry of Health dataset (21, 33). The following similar features were used: gender, age category (above 60 counted as 1 and below 60 as zero), cough details (dry cough counted as 1 and others as zero), shortness of breath, headache, sore throat, and body temperature (considered as positive if fever was above 37.5°), making it possible to compare our results with the study presented by Zoabi et al. (21).

**Figure 1:**
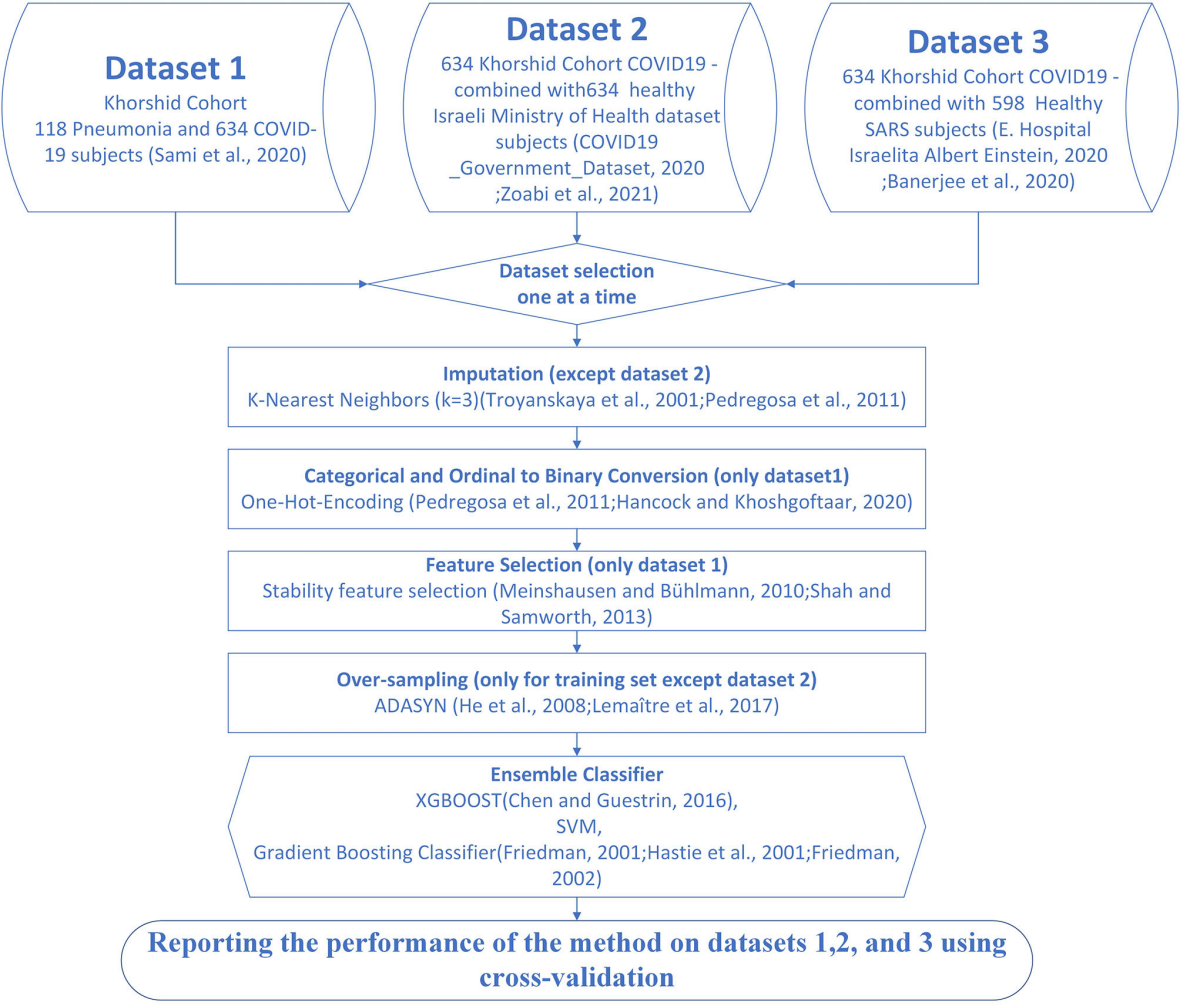
Flow of the proposed method for detection of patients with coronavirus disease-2019 (COVID-19). Dataset 1 is the Khorshid COVID-19 Cohort (KCC) dataset. Dataset 2 is the combination of 600 subjects with COVID-19 subjects from the KCC dataset and 634 healthy subjects from the Israeli Ministry of Health (IHM) dataset (21, 33). Dataset 3 is the combination of 634 subjects with COVID-19 from the KCC dataset and 598 healthy subjects from the Israelita Albert Einstein Hospital (29, 34).

We also prepared the third dataset by mixing 634 subjects with COVID-19 to KCC with 598 healthy subjects from the Israelita Albert Einstein Hospital (29, 34). The following similar features were used: WBC, PLT, CRP, LDH, Neut, and Lymph, making it possible to compare our results with the study presented by Banerjee et al. (29). Figure 1 illustrates the analyzed datasets and the proposed algorithm.

### Method

The first step toward processing data is either removing samples with missing values or imputing the missing values. For datasets 1 and 3, imputation was necessary because dropping samples with few missing values could dramatically decrease the size of the sample. The randomness of the missing data was analyzed before imputation. One of the most common imputation methods is the k-Nearest Neighbors algorithm (38, 39). The neighboring samples were set to 3 to restrict copying to the closest similarity (Figure 1). The mean, median, and mode of the features of neighboring samples were used to impute missing values in the interval, ordinal, and nominal features (40).

To make each category more comparable and avoid prioritization, we replaced each categorical or ordinal column with its binary encoded feature matrix known as one-hot encoding (39, 41). The categorical features were then converted into numerical variables by label encoding to perform standard pattern recognition techniques.

Taking informative, discriminating, and stable features is vital for devising a classification model (42). The stability selection algorithm (42, 43), which we used in this study, provided bootstrap data batches and used a baseline feature selection algorithm. Then, the stability score for each feature was calculated using the results of each bootstrap sample. In each bootstrap step, we used logistic regression (44) to analyze feature importance. In the end, features that improved the classification accuracy in most of the iterations were selected as stable features.

Datasets 1 and 3 had imbalance, and minority classes needed to be over-sampled only for the training procedure. We used the ADASYN (45, 46) over-sampling method that uses weighted distribution base on their learning difficulty. Without over-sampling, the classifiers tend to ignore the minority class, and this means that the cost function gets smaller only by fitting to the majority class.

The ensemble model consisted of three voter classifiers, XGBOOST, SVM, and gradient boosting classifier (47–49) (Figure 1). Ensemble learning is a common way of looking at a problem from different perspectives, predicting an outcome from different approaches, and combining their predictions. In some machine learning cases, only one method might have a significant error in parts of the dataset (50); ensemble learning brings more generality and robustness to the mechanism of prediction. XGBOOST (51) is an enhanced version of gradient boosted decision trees (52), designed to increase speed and performance by adding multiple regression trees and a stochastic gradient boosting mechanism. SVM (53) maps data to higher dimensions to find a hyperplane to classify samples.

We trained each model independently on the training set during cross-validation. Thus, for every single test input, we had three outputs from the classifiers. Consequently, the class with the majority of the votes was the output of the ensemble model. The hyperparameters of the models were tuned with the grid search method in Scikit-Learn (39) on the training set. The training set was split into estimation and validation with internal 5-fold cross-validation to tune the free parameters. The number of gradient boosting trees ranged between 200 and 1,200, and the objective binary logistic function and AUC evaluation metric were used for XGBOOST. The deviance loss function and the Friedman Mean Square Error metric were used for the gradient boost classifier, while the number of gradient boosting trees ranged between 200 and 1,600. For the non-linear SVM classifier, the radial basis function (RBF) kernel was used, and the penalty and kernel parameters were tuned during internal cross-validation. Noted that the objective or cost functions were minimized for the XGBOOST or gradient boost classifier, and the evaluation metric was used as the early stopping criterion.

### Evaluation

We evaluated all the models with the 10-fold stratified cross-validation method using the stratified K-fold (39). We performed linear discriminant analysis (LDA) as a base classifier and compared the performance of the proposed method with that of the LDA classifier on different datasets. We used the cross-validated confusion matrix instead of the confusion matrix of each fold to ensure that all the results were without systematic bias and error with the small dispersion index. In this study, we reported the following indices to analyze the performance of the classifiers: true positive (TP), subjects with COVID-19 correctly identified; true negative (TN), subjects without COVID-19 correctly identified; false positive (FP), subjects without COVID-19 incorrectly identified; and false negative (FN), COVID-19 incorrectly identified;

The following performance indices were then calculated:

Sensitivity or recall (Se or Rl, Equation 1); specificity (Sp, Equation 2); precision (Pr, Equation 3); area under the receiver operating characteristic (AUC, Equation 4) (54); accuracy (ACC, Equation 5); Mathew Correlation Coefficient (MCC, Equation 6; a.k.a., phi coefficient) (55), the association between the ground truth and the predicted class labels; F_1_S (F_1_ score) as the harmonic mean of the sensitivity and precision (Equation 7); Kappa [*K*(*c*)] (56), agreement rate between the ground truth and the predicted class labels (Equation 8); diagnosis odds ratio (DOR; Equation 9); and discriminant power (DP; Equation 10) (57, 58). The reference intervals of the indices AUC, Kappa, MCC, and DP were provided by Marateb et al. (59) (Supplementary Material S3).


(1) $$\begin{array}{l} Se=\frac{TP}{TP+FN}\; \end{array}$$

(2) $$\begin{array}{l} Sp=\frac{TN}{TN+FP} \end{array}$$

(3) $$\begin{array}{l} \mathrm{Pr}=\;\frac{TP}{TP+FP} \end{array}$$

(4) $$\begin{array}{l} AUC=\frac{Se+Sp}{2} \end{array}$$

(5) $$\begin{array}{l} ACC=\;\frac{TP+TN}{TP+TN+FP+FN} \end{array}$$

(6) $$\begin{array}{l} MCC=\frac{TP\times TN-FP\times FN}{\sqrt{\left(TP+FP\right)\times \left(TP+FN\right)\times \left(TN+FP\right)\times \left(TN+FN\right)}} \end{array}$$

(7) $$\begin{array}{l} F_{1}S=\frac{2\times Pr\times Se}{Pr+Se} \end{array}$$

(8) $$\begin{array}{l} K\left(c\right)=\frac{2\times \left(TP\times TN-FP\times FN\right)}{\left(TP+FP\right)\times \left(FP+TN\right)+\left(TP+FN\right)\times \left(FN+TN\right)} \end{array}$$

(9) $$\begin{array}{l} DOR=\frac{TP\times TN}{FP\times FN} \end{array}$$

(10) $$\begin{array}{l} DP=\left(\sqrt{\frac{3}{\pi }}\right)\times log\left(DOR\right) \end{array}$$

Following the Standards for Reporting Diagnostic Accuracy (STARD) (60) and Transparent Reporting of a multivariable prediction model for Individual Prognosis or Diagnosis (TRIPOD) (61) guidelines, the Confidence Interval (CI) 95% of the performance indices were provided. It showed the precision of the indices and indicated how the prediction from the analyzed samples could be generalized to the entire population. Moreover, a diagnosis system was considered clinically reliable (59, 62) if the entire following conditions were met: The sensitivity higher than 80%, the specificity, and precision higher than 95% (63, 64), and DOR higher than 100 (65).

### Statistical Analysis

Results were reported as frequencies (for categorical variables) and mean ± standard deviation (for interval variables). For interval features, those normally distributed in analyzed two classes, Independent Samples *t*-test was performed. Otherwise, and in ordinal features, the Mann–Whitney *U*-test was utilized. The pairwise χ^2^ analysis was performed for nominal features, and when the Cochran conditions were not met, the Fisher exact test was performed. The McNemar's test (66, 67) was performed to compare the performance of the proposed and the LDA classifiers on different datasets. *P*-values <0.05 were considered significant. The statistical analysis was performed using the IBM SPSS^®^ Statistics for Windows, version 22.0, which was released in 2013 (IBM Corp., Armonk, NY, United States). The classification was performed offline using Python version 3.8.8 (Python Software Foundation, 501(c) (3) non-profit organization).

## Results

The descriptive statistics of dataset 1 are provided in Table 1 for the patients with COVID and non-COVID pneumonia. Overall, 41% of the patients were female, and the average age of the patients was 57 ± 17 (years). Fourteen percent of the patients were smoker, 36% were hypertensive, and 27% had diabetes. Based on the univariate analysis (Table 1), significant differences were found in the variables age, gender, occupation, marriage status, symptom duration, contact with confirmed COVID-19 cases, history of ischemic heart disease, hypertension, chronic obstructive pulmonary disease (COPD), chronic respiratory disease, other comorbidities, body temperature, nausea, decreased appetite, weight loss, chills, headache, diarrhea, body pain, respiratory rate, oxygen saturation, systolic blood pressure, white blood cell count, lymphocyte count, hemoglobin, LDH, Na, K, Ca, P, BUN, PH, PCO2, and ESR among the patients with COVID and non-COVID pneumonia. Other comorbidities were defined as having comorbidities except for hypertension, diabetes, heart failure, percutaneous coronary intervention (PCI), coronary artery bypass graft (CABG), ischemic heart disease (IHD), cardiovascular disease (CVD), chronic respiratory diseases (CRDs), COPD, being immunocompromised, cancer, chronic kidney disease (CKD), having organ transplantation, hyperkeratosis lenticularis perstans (HLP), end-stage renal disease (ESRD), and hypothyroidism.

**Table 1:** Descriptive statistics of dataset 1.

	**Non-COVID pneumonia (*N* = 118)**	**COVID-19 pneumonia (*N* = 634)**	***P*-value**	**OR (CI 95%)**
Age (years)				
Mean (SD)	61.7 (18.3)	57.0 (15.4)	0.009	–
Gender				
Male	56 (47.5%)	389 (61.4%)	0.007	1.76 1.18–2.61
Female	62 (52.5%)	245 (38.6%)		
Occupation				
Yes	41 (34.7%)	326 (51.4%)	0.001	1.99 1.32–2.99
No	77 (65.3%)	308 (48.6%)		
Marriage status				
Single	10 (8.5%)	21 (3.3%)	0.035	–
Married	107 (90.7%)	608 (95.9%)		–
Divorced or widowed	1 (0.8%)	5 (0.8%)		–
Symptom's duration (day)				
Mean (SD)	6.11 (6.04)	7.97 (5.71)	0.002	–
Smoking status				
Yes	23 (19.5%)	82 (12.9%)	0.081	0.61 0.37–1.02
No	95 (80.5%)	552 (87.1%)		
Contact with confirmed COVID-19 cases				
Yes	4 (3.4%)	95 (15.0%)	0.001	5.02 1.81–13.94
No	114 (96.6%)	539 (85.0%)		
Quarantine before admission				
Yes	110 (93.2%)	589 (92.9%)	1	0.95 0.44–2.07
No	8 (6.8%)	45 (7.1%)		
**Comorbidities**				
Ischemic heart disease				
Yes	27 (22.9%)	91 (14.4%)	0.028	0.56 0.35–0.92
No	91 (77.1%)	543 (85.6%)		
Hypertension				
Yes	53 (44.9%)	221 (34.9%)	0.048	0.66 0.44–0.98
No	65 (55.1%)	413 (65.1%)		
Chronic kidney disease				
Yes	5 (4.2%)	18 (2.8%)	0.604	0.66 0.24–1.81
No	113 (95.8%)	616 (97.2%)		
COPD				
Yes	10 (8.5%)	13 (2.1%)	<0.001	0.23 0.10–0.53
No	108 (91.5%)	621 (97.9%)		
Cancer				
Yes	3 (2.5%)	15 (2.4%)	1	0.93 0.26–3.26
No	115 (97.5%)	619 (97.6%)		
Chronic respiratory disease				
Yes	27 (22.9%)	56 (8.8%)	<0.001	0.33 0.20–0.54
No	91 (77.1%)	578 (91.2%)		
Other comorbidities				
Yes	47 (39.8%)	143 (22.6%)	<0.001	0.44 0.29–0.66
No	71 (60.2%)	491 (77.4%)		
CCI				
Mean (SD)	–	2.25 (2.10)	–	–
**Signs and symptoms**				
Sneeze				
Yes	4 (3.4%)	56 (8.8%)	0.069	2.76 0.98–7.77
No	114 (96.6%)	578 (91.2%)		
Runny nose				
Yes	10 (8.5%)	73 (11.5%)	0.419	1.41 0.70–2.81
No	108 (91.5%)	561 (88.5%)		
Body temperature (°C)				
Mean (SD)	37.1 (1.10)	37.5 (1.10)	<0.001	–
Fatigue				
Yes	73 (61.9%)	482 (76.0%)	0.002	1.95 1.29–2.96
No	45 (38.1%)	152 (24.0%)		
Cough details				
Other	41 (34.7%)	117 (18.5%)	<0.001	0.43 0.28–0.65
Dry	77 (65.3%)	517 (81.5%)		
Nausea				
Yes	43 (36.4%)	269 (42.4%)	0.267	1.29 0.86–1.93
No	75 (63.6%)	365 (57.6%)		
Decreased appetite				
Yes	70 (59.3%)	508 (80.1%)	<0.001	2.76 1.82–4.19
No	48 (40.7%)	126 (19.9%)		
Weight loss				
Yes	27 (22.9%)	268 (42.3%)	<0.001	2.47 1.56–3.90
No	91 (77.1%)	366 (57.7%)		
Chills				
Yes	64 (54.2%)	457 (72.1%)	<0.001	2.18 1.46–3.26
No	54 (45.8%)	177 (27.9%)		
Headache				
Yes	39 (33.1%)	306 (48.3%)	0.003	1.89 1.25–2.86
No	79 (66.9%)	328 (51.7%)		
Shortness of breath				
Yes	83 (70.3%)	440 (69.4%)	0.925	0.96 0.62–1.47
No	35 (29.7%)	194 (30.6%)		
Influenza vaccine				
Yes	8 (6.8%)	22 (3.5%)	0.153	0.49 0.21–1.14
No	110 (93.2%)	612 (96.5%)		
Diabetes				
Yes	32 (27.1%)	172 (27.1%)	1	1.00 0.64–1.56
No	86 (72.9%)	462 (72.9%)		
Diarrhea				
Yes	21 (17.8%)	200 (31.5%)	0.004	2.13 1.29–3.51
No	97 (82.2%)	434 (68.5%)		
Sore throat				
Yes	21 (17.8%)	134 (21.1%)	0.484	1.24 0.74–2.06
No	97 (82.2%)	500 (78.9%)		
Vomiting				
Yes	27 (22.9%)	178 (28.1%)	0.293	1.32 0.83–2.09
No	91 (77.1%)	456 (71.9%)		
Abdominal pain				
Yes	19 (16.1%)	123 (19.4%)	0.476	1.25 0.74–2.13
No	99 (83.9%)	511 (80.6%)		
Body pain				
Yes	59 (50.0%)	446 (70.3%)	<0.001	2.37 1.59–3.54
No	59 (50.0%)	188 (29.7%)		
**Vital symptoms**				
Pulse rate (per min)				
Mean (SD)	98.1 (20.3)	96.0 (16.4)	0.294	–
Respiratory rate (per min)				
Mean (SD)	23.2 (5.48)	21.9 (5.19)	0.019	–
Oxygen saturation (%)				
Mean (SD)	86.8 (8.39)	89.3 (7.19)	0.003	–
Systolic blood pressure (mmHg)				
Mean (SD)	139 (24.4)	133 (19.5)	0.016	–
Diastolic blood pressure (mmHg)				
Mean (SD)	83.1 (15.3)	82.0 (28.5)	0.559	–
**Laboratory findings**				
White blood cell count (×10e9/L)				
Mean (SD)	9.71 (8.16)	6.13 (3.01)	<0.001	–
Neutrophil count (×10e9/L)				
Mean (SD)	7.51 (14.5)	7.35 (11.2)	0.247	–
Lymphocyte count (×10e9/L)				
Mean (SD)	1.76 (11.10)	2.07 (9.65)	0.006	–
Platelet count (×10e9/L)				
Mean (SD)	203 (91.7)	191 (70.9)	0.186	–
Hemoglobin (g/dL)				
Mean (SD)	12.7 (2.48)	13.3 (1.75)	0.018	–
CRP (mg/L)				
Mean (SD)	35.2 (21.9)	35.3 (17.4)	0.978	–
LDH (U/L)				
Mean (SD)	1,100 (1,050)	886 (592)	0.035	–
AST (U/L)				
Mean (SD)	60.4 (171)	46.6 (46.3)	0.386	–
ALT (U/L)				
Mean (SD)	60.1 (290)	33.9 (44.5)	0.328	–
ALP (U/L)				
Mean (SD)	192 (88.7)	177 (96.0)	0.101	–
Na (meq/L)				
Mean (SD)	136 (4.13)	135 (3.38)	0.011	–
K (mmol/L)				
Mean (SD)	3.92 (0.59)	3.78 (0.42)	0.019	–
Ca (mg/dL)				
Mean (SD)	8.72 (0.88)	8.53 (0.70)	0.030	–
P (mg/dL)				
Mean (SD)	3.35 (1.21)	2.94 (0.73)	<0.001	–
Mg (mg/dL)				
Mean (SD)	1.94 (0.22)	1.95 (0.24)	0.914	–
BUN (mg/dL)				
Mean (SD)	23.80 (18.00)	19.50 (13.60)	0.016	–
Cr (mg/dL)				
Mean (SD)	1.43 (1.43)	1.20 (0.99)	0.093	–
PH				
Mean (SD)	7.33 (0.09)	7.36 (0.06)	<0.001	–
PCO2 (mmHg)				
Mean (SD)	50.6 (15.8)	44.4 (9.38)	<0.001	–
ESR (mm/h)				
Mean (SD)	42.3 (28.1)	48.0 (26.4)	0.045	–

*OR, odds ratio; CI, confidence interval*.

The descriptive statistics of dataset 2 are provided in Table 2 for the patients with COVID-19 and control subjects. All the variables had significant differences between the patients with COVID-19 and control subjects. Since the variables of the control group of dataset 3 were normalized (29, 34) and the original values were not available, a descriptive table was not provided for dataset 3.

**Table 2:** Descriptive statistics of the dataset 2.

	**Non-COVID (*N* = 634)**	**COVID-19 (*N* = 634)**	***P*-value**	**OR (CI 95%)**
Age category (years)				
>60	89 (14.0%)	276 (43.5%)	<0.001	4.72 3.59–6.21
≤ 60	545 (86.0%)	358 (56.5%)		
Gender				
Male	291 (45.9%)	389 (61.4%)	<0.001	1.87 1.50–2.34
Female	343 (54.1%)	245 (38.6%)		
Cough category				
Other	600 (94.6%)	117 (18.5%)	<0.001	0.01 0.01–0.02
Dry	34 (5.4%)	517 (81.5%)		
Shortness of breath				
Yes	1 (0.2%)	440 (69.4%)	<0.001	–
No	633 (99.8%)	194 (30.6%)		
Headache				
Yes	1 (0.2%)	306 (48.3%)	<0.001	–
No	633 (99.8%)	328 (51.7%)		
Sore throat				
Yes	3 (0.5%)	134 (21.1%)	<0.001	56.37 17.84–178.06
No	631 (99.5%)	500 (78.9%)		
Fever (body temperature > 37.5°C)				
Yes	14 (2.2%)	308 (48.6%)	<0.001	41.84 24.09–72.68
No	620 (97.8%)	326 (51.4%)		

The selected features of datasets 1, 2, and 3, and their importance (weights between zero and 1) by the proposed algorithm are listed in Table 3. The most important features were WBC, shortness of breath, and CRP in datasets 1, 2, and 3, respectively.

**Table 3:** Selected features of datasets 1, 2, and 3, along with their importance (shown as weights between zero and 1) using the proposed algorithm.

**Dataset 1**		**Dataset 2**		**Dataset 3**	
**Feature**	**Weight**	**Feature**	**Weight**	**Feature**	**Weight**
WBC	1.000	Shortness of breath	1.000	CRP	1.000
PCO2	0.995	Cough details	0.539	WBC	0.322
Decreased appetite	0.957	Headache	0.111	LDH	0.215
Contact with conformed COVID-19 patients	0.947	Body temperature	0.034	Neutrophils	0.158
Cough details	0.914	Age	0.026	PLT	0.157
Other comorbidities	0.901	Sore throat	0.025	Lymphocytes	0.127
*P*	0.882	Gender	0.004		
Myalgia	0.862	
Body temperature	0.846	
Chronic respiratory disease	0.825	
Symptom duration	0.819	
COPD	0.782	
Occupation	0.780	
Gender	0.777	
Na	0.775	
LDH	0.772	
HB	0.748	
Weight loss	0.747	
Chills	0.709	
Diarrhea	0.674	
ESR	0.625	
SatO2	0.5928	

The performance of the proposed algorithm and the LDA classifier on datasets 1, 2, and 3 is shown in Table 4. Such indices were provided in the cross-validated confusion matrix. The system is clinically reliable on datasets 1 and 2, while it is not reliable on dataset 3 because of a Type I error of 0.1, false discovery rate (FDR) of 0.09, and DOR of 67.8. It showed excellent balanced diagnosis accuracy (AUC ≥ 0.9), excellent class labeling agreement rate with the PCR test [K(c) ≥ 0.75], high/very high correlation between predicted and observed class labels (MCC ≥ 0.7), and fair discriminant power (3> DP ≥ 2) for datasets 1 and 2. Note that the performance of the proposed algorithm was significantly better than that of the LDA classifier on datasets 1 (*P* < 0.001), 2 (*P* < 0.01), and 3 (*P* < 0.001). Moreover, the average performance of the proposed algorithm and the LDA classifier on the test folds is provided in Table 5, showing the reproducibility of the results.

**Table 4:** Performance of the proposed algorithm and linear discriminant analysis (LDA) classifier on datasets 1, 2, and 3, based on the results of cross-validated confusion matrix.

**Dataset**	**Classifier**	**Indices**	**Se**	**Sp**	**PPV**	**NPV**	**AUC**	**MCC**	**F_**1**_S**	**DOR**	**DP**	**K(c)**
Dataset 1	Proposed	Value	0.96	0.95	0.99	0.82	0.96	0.87	0.98	495.88	2.63	0.86
		CI 95%	0.94–0.98	0.90–0.99	0.98–1	0.76–0.89	0.94–0.97	0.85–0.88	0.95–1	197.46–1245.30	2.24–3.02	0.81–0.91
	LDA	Value	0.81	0.61	0.92	0.37	0.71	0.35	0.86	6.57	0.80	0.33
		CI 95%	0.78–0.84	0.52–0.70	0.89–0.94	0.30–0.44	0.66–0.75	0.28–0.41	0.83–0.89	4.32–9.99	0.62–0.98	0.24–0.42
Dataset 2	Proposed	Value	0.96	0.97	0.97	0.97	0.97	0.94	0.97	952.00	2.91	0.94
		CI 95%	0.95–0.98	0.96–0.98	0.96–0.98	0.95–0.98	0.96–0.98	0.93–0.94	0.96–0.98	505.57–1792.64	2.64–3.18	0.91–0.96
	LDA	Value	0.94	0.97	0.97	0.94	0.95	0.90	0.95	422.20	2.57	0.90
		CI 95%	0.92–0.96	0.95–0.98	0.95–0.98	0.92–0.95	0.94–0.96	0.90–0.91	0.94–0.97	246.54–722.99	2.34–2.80	0.88–0.93
Dataset 3	Proposed	Value	0.94	0.91	0.90	0.94	0.92	0.85	0.92	156.35	2.14	0.85
		95% CI	0.92–0.96	0.89–0.93	0.88–0.93	0.93–0.96	0.91–0.94	0.83–0.86	0.90–0.94	101.03–241.33	1.96–2.33	0.82–0.88
	LDA	Value	0.56	0.55	0.39	0.71	0.56	0.11	0.46	1.60	0.20	0.10
		CI 95%	0.51–0.61	0.52–0.59	0.35–0.43	0.68–0.75	0.52–60	0.01–0.17	0.42–0.50	1.26–2.02	0.10–0.30	0.05–0.16

*CI, confidence interval; Se, sensitivity; Sp, specificity; PPV, positive predictive value; NPV, negative predictive value; AUC, area under the ROC; MCC, Matthews Correlation Coefficient; F_1_S, F_1_ score; DOR, diagnostic odds ratio; DP, discriminant power; K(c), Cohen's Kappa*.

**Table 5:** Performance of the proposed algorithm and LDA classifier on datasets 1, 2, and 3 over the test folds during cross-validation in mean ± standard deviation (SD).

**Dataset**	**Indices classifier**	**Se**	**Sp**	**PPV**	**NPV**	**AUC**	**MCC**	**F_**1**_S**	**DOR**	**DP**	**K(c)**
Dataset 1	Proposed	0.96 ± 0.02	0.95 ± 0.04	0.99 ± 0.01	0.83 ± 0.06	0.96 ± 0.02	0.87 ± 0.05	0.98 ± 0.01	423.18 ± 279.76	2.51 ± 0.30	0.86 ± 0.05
	LDA	0.79 ± 0.02	0.62 ± 0.03	0.92 ± 0.01	0.35 ± 0.03	0.70 ± 0.02	0.33 ± 0.04	0.85 ± 0.01	6.17 ± 1.35	0.77 ± 0.09	0.31 ± 0.04
Dataset 2	Proposed	0.96 ± 0.02	0.97 ± 0.02	0.97 ± 0.02	0.96 ± 0.02	0.97 ± 0.01	0.94 ± 0.02	0.97 ± 0.01	1050.30	2.95 ± 0.07	0.94 ± 0.02
	LDA	0.97 ± 0.02	0.94 ± 0.03	0.94 ± 0.02	0.97 ± 0.02	0.95 ± 0.01	0.90 ± 0.03	0.95 ± 0.01	440.60 ± 29.35	2.58 ± 0.03	0.90 ± 0.03
Dataset 3	Proposed	0.90 ± 0.04	0.94 ± 0.02	0.94 ± 0.02	0.91 ± 0.03	0.92 ± 0.02	0.85 ± 0.04	0.92 ± 0.2	258.38 ± 328.30	2.20 ± 0.31	0.85 ± 0.04
	LDA	0.39 ± 0.06	0.71 ± 0.08	0.57 ± 0.07	0.55 ± 0.03	0.55 ± 0.04	0.11 ± 0.09	0.46 ± 0.6	1.78 ± 0.82	0.20 ± 0.18	0.11 ± 0.09

*CI, confidence interval; Se, sensitivity; Sp, specificity; PPV, positive predictive value; NPV, negative predictive value; AUC, area under the ROC; MCC, Matthews Correlation Coefficient; F_1_S, F_1_ score; DOR, diagnostic odds ratio; DP, discriminant power; K(c), Cohen's Kappa*.

Also, the random accuracy of the datasets was estimated by random assignment of classes, considering the prevalence of the minority class. Ten thousand simulations were performed. The range (mean) of the obtained random accuracies was 68–79 (74%), 43–56 (50%), and 44–56% (50%) for datasets 1, 2, and 3, respectively. The obtained accuracy of the proposed method and the LDA classifier was 96, 78 (dataset 1), 97, 96 (dataset 2), and 93, 56% (dataset 3) on the cross-validated confusion matrix. Except for the results of the LDA classifier on datasets 1 and 3, the other results were higher than the random classification thresholds.

## Discussion

During the pandemic, morbidity and mortality can be reduced by early prediction of population infection risks, ensuring efficient treatment planning and resource allocation. A high patient load is prevented by rapid disease diagnosis. Higher mortality rates are an essential consequence of an overloaded medical system due to inefficient management of limited medical resources.

This study constructed a prediction model trained using the cohort in KCC. It accurately forecasted infection cases in comparison with both pneumonia (dataset 1) and healthy subjects (datasets 2 and 3) (Table 4). There are some studies in the literature for COVID-19 diagnosis (68). However, to the best of our knowledge, no similar study was performed to classify COVID-19 and non-COVID pneumonia, without using image processing methods on CT-scan results. Moreover, the cross-validation method used in our study guarded against testing hypotheses suggested by the data [type III errors (69)].

The combination of the PCR and CT-scan results was used as the ground truth in our study. It was shown that the PCR test is not 100% correct to be considered as gold standard (70). Mainly, it was shown to have a false negative rate (FNR) ranging from 0.018 to 0.58, with a median of 0.11 (71), in addition to a sensitivity of 83.3% (15). It was recommended to combine the results of PCR and CT-scan with improving the ground truth for COVID-19, mainly to improve the FNR of the PCR test (72, 73).

Notably, three different datasets related to control individuals in comparison with our COVID-19 dataset were observed in this analysis.

In the first round, COVID-19 diagnosis compared with pneumonia model performance showed very good results using 22 pre-admission and hospital-based characteristics (Table 4). The analyses of this study highlighted three non-invasive features: WBC (weight of 1), PCO2 (0.99), and contact with confirmed COVID-19 cases (0.95).

However, CT scan has become the primary gold standard for screening COVID-19 cases; however, it cannot be used to identify specific viral infections (74). Furthermore, some patients with COVID-19 can also present with standard CT imaging in the early stage (74). Thus, clinical symptoms, pre-admission variables, and laboratory tests can be more specific for early COVID-19 infection. According to studies, the most common early symptoms of COVID-19 were cough, fever, myalgia, and diarrhea (75). The results of weighted features in our study presented that cough (w = 0.91) and myalgia (w = 0.86) were the two most essential symptom predictors after decreased appetite.

There are some studies in the literature that used similar features for COVID-19 diagnosis. Long et al. (6) reported that WBC and body temperature were good factors in uncovering the COVID-19 infection. Another study by Brinati et al. (18) also showed that LDH and WBC were essential features. Moreover, Gongj and Qiu (76) illustrated that LDH was one of the good features for predicting severe COVID-19. Likewise, Goodman-Meza et al. (19) reported gender, HB, and LDH as essential features related to COVID-19 infection.

In the second round, we developed our predictive models for diagnosing patients with COVID-19 and healthy control subjects with the eight standard non-invasive features used in Zoabi et al. (21). Our joint model provides rapid and accurate predictions using seven features. While shortness of breath (w = 1) and cough details (w = 0.54), were the most critical features in our analysis for dataset 2 (Table 3), cough details and fever were the essential features in Zoabi et al. (21). In fact, the performance of the proposed method was higher in dataset two compared with dataset 1 in almost all the indices (Table 4), knowing that only eight demographic and symptom features were used in dataset 2 compared with the 22 selected features in dataset 1 (Table 3). It primarily showed that the classification of COVID-19 was more difficult with non-COVID pneumonia (dataset 1) compared with healthy control subjects (dataset 2). In fact, when we used reduced feature sets for dataset 1, the performance significantly dropped. Also, comparing datasets 1 and 2, shortness of breath and sore throat were not statistically significant between COVID-19 and non-COVID-19 pneumonia (Table 1), while they were statistically significant between COVID-19 and healthy control subjects (Table 2). It showed the similarity of the characteristics of dataset 1 compared with dataset 2 for standard features.

In the last round, using another dataset related to six invasive laboratory variables (29, 34), our results revealed that invasive features showed an overall good prediction capacity between COVID-19 patients and healthy people (Table 4). However, the results were not as good as those obtained on datasets 1 and 2, showing that symptoms added valuable information to blood tests for screening. Moreover, among the six variables, neutrophil, platelet counts, and CRP were not statistically significant in dataset 1 (Table 1), showing that patients with COVID-19 and non-COVID-19 pneumonia showed similar features based on 50% of the laboratory variables used in dataset 3. This difference in prediction criteria reached lower sensitivity, specificity, and AUC using invasive characteristics compared with the model using non-invasive variables. It might be because invasive biomarkers have a distinct temporal dynamic behavior (13).

Both CT-scan and laboratory-based methods share the main limitation when applied to the population. For cases such as COVID-19 infection, where the prevalence of the disease (i.e., the minority class) is low in the population, the performance of the diagnosis methods drops. It happens especially when the analyzed imbalanced test datasets are balanced. For example, Sun et al. designed a diagnosis method based on CT-scan image processing and reached 93 and 90% sensitivity and specificity, respectively, to discriminate between COVID and non-COVID pneumonia (25). Considering the prevalence of 14% of COVID-19 (77), it is possible to use Bayes' theorem to predict the true PPV and NPV of the diagnosis method when applied to the population (Equations 11, 12) (78):


(11) $$\begin{array}{l} PPV=\frac{Se\times P}{Se\times P+\left(1-Sp\right)\times \left(1-P\right)} \end{array}$$

(12) $$\begin{array}{l} NPV=\frac{Sp\times \left(1-P\right)}{Sp\times \left(1-P\right)+\left(1-Se\right)\times P} \end{array}$$

where *P* is the prevalence of COVID-19. The parameter PPV is also the probability of having COVID-19 when the proposed diagnosis test is positive. Similarly, NPV is the probability of not having COVID-19 when the proposed diagnosis test is negative. For this example, the estimated PPV and NPV were 60 and 99%. When examining our proposed method, the true PPV and NPV were 75 and 99%. Thus, the performance of the proposed method must be improved to be used in clinical practice. Noted that such a problem is similar to many other areas in which the prevalence of the minority class is very low (79).

Indeed, key laboratory features, such as LDH, CRP, WBC, and PLT, have high temporal dynamicity, and in a relatively short time, they rise and return to their normal range (80). Additionally, laboratory variable abnormalities only show disruptions in body systems linked to many infectious diseases. In contrast, many non-invasive features, such as symptoms and age, contain a substantial amount of less dynamic data.

We proposed that a prediction model can be used for risk assessment to notify high-risk subjects for receiving the complementary RT-PCR test. A promising area for future research is to analyze the combined performance of the new rapid clinical application diagnosis system, machine learning algorithms, and new biomarkers (81).

Nevertheless, this study still had several limitations. First, the major participants were from Isfahan province. Furthermore, nationwide studies are needed to access the best generality of the suggested model. Moreover, because of the inaccessibility of the data of healthy control subjects, control subjects from other studies (21, 29) were also used in datasets 2 and 3. The KCC dataset was mainly limited to alpha variants of COVID-19 (32), and it is necessary to perform extra validation on other mutants to assess its performance (82). Also, preparing the COVID-19 diagnosis risk chart, which is a valuable tool, is the focus of our feature study. Finally, the RT-PCR and chest CT-scan used as gold standard are not 100% accurate, and the agreement rate (Cohen's Kappa) reported in the study is, thus, a better index than traditional predictive indices (83).

## Conclusion

In conclusion, we designed a reliable computer-aided diagnosis system to classify patients with COVID-19 from non-COVID pneumonia. Demographics, symptoms, and blood tests were used in the proposed system. The proposed system is a promising screening tool for COVID-19.

## Data Availability Statement

The datasets presented in this study can be found in online repositories. The names of the repository/repositories and accession number(s) can be found at: https://doi.org/10.6084/m9.figshare.16682422.

## Ethics Statement

The studies involving human participants were reviewed and approved by the Ethics Committee of the Isfahan University of Medical Sciences (IUMS). The patients/participants provided their written informed consent to participate in this study.

## Author Contributions

HM, FZ, MRM, RS, MM, MAM, MW, and HB participated in conceptualization, investigation, and methodology. HM, FZ, and MRM participated in visualization, software, and validation. RS, SHJ, MM, MAM, MW, and HB participated in project administration. HM, FZN, MRM, and MM participated in formal analysis. RS, SHJ, MM, FDN, and MA-S participated in data acquisition. FDN, MA-S, and RS participated in data curation. RS, SHJ, MM, and MAM participated in funding acquisition. MM, MAM, and RS participated in supervision. MM, RS, HB, MAM, and MW participated in the interpretation of the results. HM, FZN, MRM, MM, and MW participated in writing—original draft, while RS, SHJ, FDN, MA-S, MAM, and HB participated in writing—review and editing. All authors read and approved the final manuscript and agreed to be accountable for all aspects of this study.

## Funding

The research that led these results has received funding from the European Union's Horizon 2020 Research and Innovation Programme under the Marie Skłodowska-Curie Grant Agreement No. 712949 (TECNIOspring PLUS), and from the Agency for Business Competitiveness of the Government of Catalonia (TECSPR18-1–0017). The TECSPR18-1–0017 project provided the APC. These funders had no role in study design, data collection, analysis, decision to publish, or manuscript preparation.

## Conflict of Interest

The authors declare that the research was conducted in the absence of any commercial or financial relationships that could be construed as a potential conflict of interest.

## Publisher's Note

All claims expressed in this article are solely those of the authors and do not necessarily represent those of their affiliated organizations, or those of the publisher, the editors and the reviewers. Any product that may be evaluated in this article, or claim that may be made by its manufacturer, is not guaranteed or endorsed by the publisher.

## Abbreviations

ACC: Accuracy
ALT: Alanine Transaminase
ALP: Alkaline Phosphatase
AUC: The area under the receiver operating characteristic
AST: Aspartate Aminotransferase
Ca: Blood Calcium
Cr: Blood Creatinine
BUN: Blood Urea Nitrogen
CCI: Charlson Comorbidity Index
HRCT: Chest computed High-resolution CT
CT: Computerized Tomography
CI: Confidence Interval
CRP: C-Reactive Protein
DOR: diagnosis odds ratio
DP: discriminant power
F1S: F1-score
FN: False Negative
FP: False Positive
GMDH: Group method of data handling
K(c): Kappa
KCC: Khorshid COVID Cohort
LDH: Lactate Dehydrogenase
WBC: Leukocytes
LightGBM: light gradient-boosting machine learning algorithm based on decision-tree
Lymph: Lymphocytes
LDA: Linear Discriminant Analysis
Mg: Magnesium
MCC: Mathew Correlation Coefficient
MLP: Multi-level Perceptron neural network
Neut: Neutrophils
PLT: Platelets
K or P: Potassium
RT-PCR: reverse-transcription polymerase chain reaction
RBF: Radial Basis Function
SES: socioeconomic status
Na: Sodium
STARD: Standards for Reporting Diagnostic Accuracy
XGBOOST: Stochastic Gradient Descent
SVM: Support Vector Machine
TRIPOD: Transparent Reporting of a multivariable prediction model for Individual Prognosis or Diagnosis
TN: True Negative
TP: True Positive
WHO: World Health Organization
α-HBDH: α-Hydroxybutyrate Dehydrogenase.
